# Activation of toll like receptor 4 (TLR4) promotes cardiomyocyte apoptosis through SIRT2 dependent p53 deacetylation

**DOI:** 10.1038/s41598-020-75301-4

**Published:** 2020-11-06

**Authors:** Parmeshwar Bajirao Katare, Hina Lateef Nizami, Bugga Paramesha, Amit K. Dinda, Sanjay K. Banerjee

**Affiliations:** 1 Drug Discovery Research Centre (DDRC), Translational Health Science and Technology Institute (THSTI), Faridabad, Haryana 121001 India; 2 Department of Pathology, All India Institute of Medical Sciences (AIIMS), Ansari Nagar, New Delhi, 110029 India; 3 National Institute of Pharmaceutical Education and Research (NIPER), Guwahati, Assam 781101 India

**Keywords:** Molecular medicine, Heart failure, Experimental models of disease

## Abstract

Cardiomyocyte inflammation followed by apoptosis and fibrosis is an important mediator for development and progression of heart failure. Activation of toll-like receptor 4 (TLR4), an important regulator of inflammation, causes the progression of cardiac hypertrophy and injury. However, the precise mechanism of TLR4-mediated adverse cardiac outcomes is still elusive. The present study was designed to find the role of TLR4 in cardiac fibrosis and apoptosis, and molecular mechanism thereof. Rats were treated with TLR4 agonist (LPS 12.5 μg/kg/day) through osmotic pump for 14 days. To simulate the condition in vitro, H9c2 cells were treated with LPS (1 μg/ml). Similarly, H9c2 cells were transfected with TLR4 and SIRT2 c-DNA clone for overexpression. Myocardial oxidative stress, inflammation, fibrosis and mitochondrial parameters were evaluated both in vitro and in vivo. Cardiac inflammation after LPS treatment was confirmed by increased TNF-α and IL-6 expression in rat heart. There was a marked increase in oxidative stress as observed by increased TBARS and decreased endogenous antioxidants (GSH and catalase), along with mitochondrial dysfunction as measured by mitochondrial complex activity in LPS-treated rat hearts. Histopathological examination showed the presence of cardiac fibrosis after LPS treatment. Protein expression of nuclear p53 and cleaved caspase-7/caspase-9 was significantly increased in LPS treated heart. Similar to in vivo study, nuclear translocation of p53, mitochondrial dysfunction and cellular apoptosis were observed in H9c2 cells treated with LPS. Our data also indicate that decreased expression of SIRT2 was associated with increased acetylation of p53 after LPS treatment. In conclusion, TLR4 activation in rats promotes cardiac inflammation, mitochondrial dysfunction, apoptosis and fibrosis. p53 and caspase 7/caspase 9 were found to play an important role in TLR4-mediated apoptosis. Our data suggest that, reducing TLR4 mediated fibrosis and apoptosis could be a novel approach in the treatment of heart failure, keeping in the view the major role played by TLR4 in cardiac inflammation.

## Introduction

The heart is an organ that works continuously with limited capacity for repair and regeneration^1^. It is vulnerable to innumerable stressful conditions and must respond to these insults by adapting to the workload demands. Many a time, these stresses may push a cardiomyocyte towards senescence. Cardiomyocyte cell death is a prominent characteristic of various cardiac diseases, including heart failure (HF) and ischemia/reperfusion (IR) injury^2^.

Cardiomyocytes are terminally differentiated myocardial cells, which rarely undergo apoptosis. However, apoptosis may increase as a result of stress and injury, progressing to adverse outcomes such as heart failure. Due to limited ability of cardiaomyocytes to regenerate, even a low level of apoptosis can have exhaustive effects on cardiac function. Mani et al. had hypothesized that 0.1% apoptosis rate can lead to approximately 37% cardiomyocyte loss over a span of a year^3^. This hypothesis was tested with cardiac myocyte-specific expression inducible caspase 8 transgenic mice. These transgenic mice developed severe, dilated cardiomyopathy (DCM) over 8 weeks and died within 2–6 months^4^. This report suggests that even a small increase in the rate of apoptosis can be very detrimental for cardiac function, and is thus, an important component of HF pathogenesis.

Several attempts have been made in past to develop drug molecules targeting apoptosis for the treatment of cardiovascular diseases. Etanercept, a recombinant human soluble protein that antagonises TNF-α receptor did not demonstrate significant clinical benefit in HF^5^. However, it was observed that TNF-α inhibition may rather adversely affect the cardiovascular disease outcome in these patients^6^. A similar trial with Infliximab failed to show any improvements in HF. Instead, Infliximab increased the adverse events in HF patients^6^. Pentoxifylline, a xanthine derivative that modulates TNF-α mRNA expression, was found to be beneficial in idiopathic dilated cardiomyopathy in animal model^7^. A large multicentre trial is warranted to further evaluate the safety and efficacy of this drug in humans.

There are several mechanisms by which apoptosis is activated in diseased heart. One of these mechanisms is toll-like receptor (TLR) 4 induced inflammation. TLRs are integral mediators of innate immunity, involved in detection of danger signals in the body either from pathogens, called pathogen associated molecular patterns (PAMPs) or from damaged host cells, called damage associated molecular patterns (DAMPs)^8^. After activation, TLRs initiate an adaptive inflammatory response, which if left unmitigated, could result in cardiac injury. Out of 13 TLRs in the body, TLR4 is found to play an important role in cardiovascular pathophysiology. TLR4 activation has been demonstrated to be critical for the development into the phase of dilated cardiomyopathy (DCM)^9^. TLR4 mediated inflammatory immune response characterised by leucocyte activation and infiltration in myocardium, initiates cytokine secretion and protease release, thereby aggravates apoptosis and necrosis of cardiomyocytes. Beside this, damaged myocardium could further release several DAMPs, including HMGB1, HSPs and mitochondrial components recognized by TLR4^10,11^. These DAMPs promote DCM formation via a positive feedback loop that aggravate cardiac inflammation and injury. Recent studies have shown that, TLR4 knockout mice were protected from doxorubicin induced cardiotoxicity in mice^12^. Whereas, targeting TLR4 or downstream effectors of TLR4 such as MyD88 showed protective effects in many heart failure models, namely, myocardial ischemia and pressure overload^13,14^. Recently, we have demonstrated that inhibition of TLR4 attenuates isoproterenol induced cardiac hypertrophy in rats^15^.

Taken together, above evidence strongly suggests that TLR4 activation acts as an important mediator for myocardial apoptosis. However, the precise mechanism of TLR4 mediated cardiac apoptosis remains elusive. Delineating the molecular basis of TLR4 in this critical effector pathway is needed to validate TLR4 as a potential target for cardiac inflammation and heart failure. Therefore, this study was undertaken to elucidate the role of TLR4 activation in cardiac apoptosis and fibrosis, and the underlying molecular mechanism.

## Results

### TLR4 activation decreased heart weight-to-tail length ratio, and triggered ECG perturbations in rat heart

**Figure 1:**
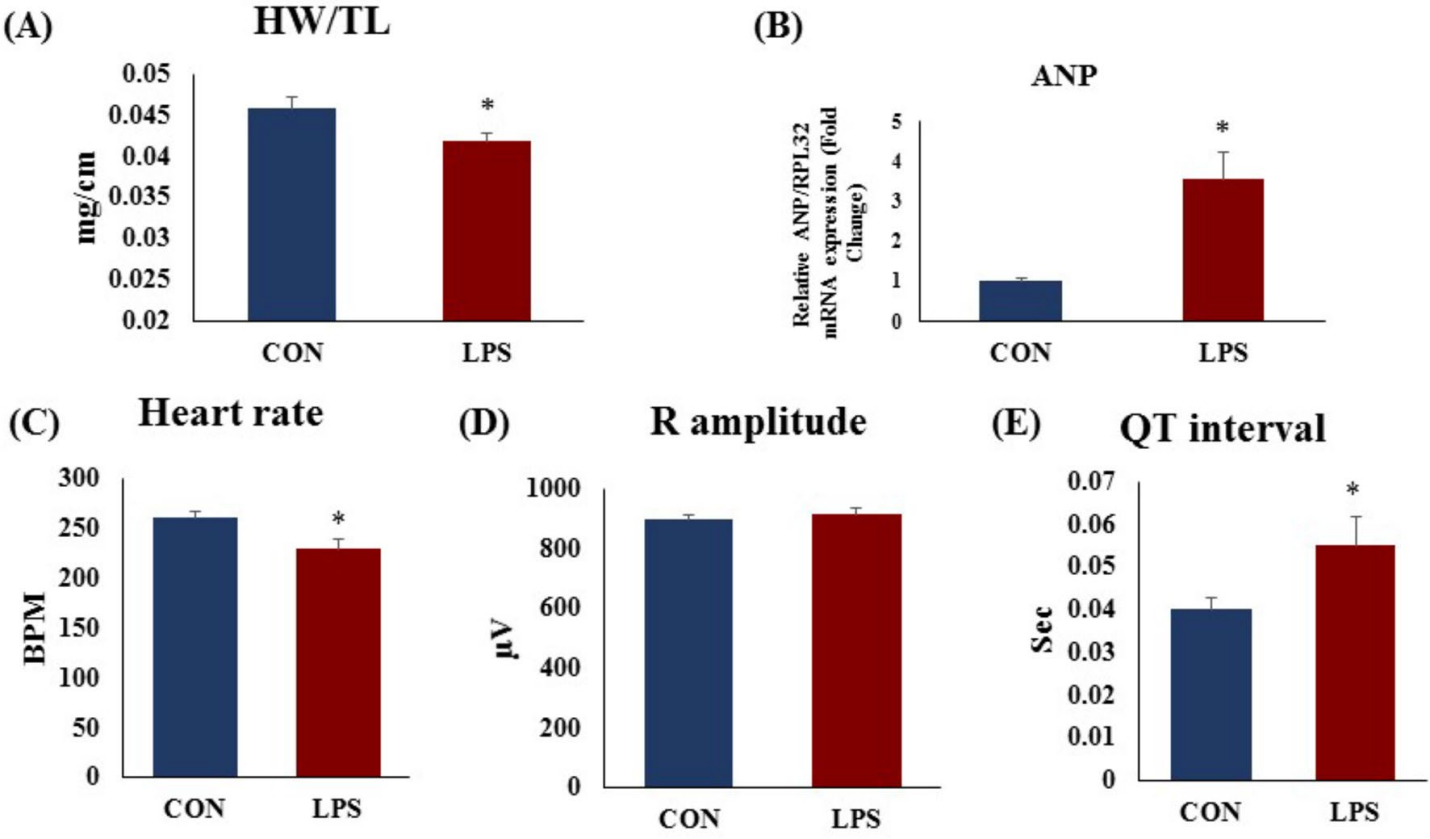
Cardiac phenotypes, mRNA expression AND Electrocardiogram (ECG) perturbations in cardiac tissue and effect of TLR 4 activation. (**A**) Heart weight to tail length ratio (HW/TL) (**B**) mRNA expression of ANP (**C**) heart rate in beats per minute (BPM) (**D**) R amplitude (**E**) QT duration. The mRNA expression data was normalized to the expression of reference gene, *ribosomal protein L32* (RPL32). Data shown as Mean ± SEM, (N = 6 for HW/TL, N = 4 for mRNA expression and ECG) *p < 0.05, **p < 0.01 vs CON.

In our study, we found that heart weight-to-tail length ratio (Fig. 1A) was decreased significantly (p < 0.05) in LPS group as compared to CON group indicating the cardiac atrophy induced after LPS treatment. Interestingly, mRNA expression of myocardial ANP (Fig. 1B) was increased in LPS group. We found decreased heart rate (Fig. 1C) and analysis of electrocardiogram showed prolonged QT interval (Fig. 1E) after TLR4 activation in LPS group. R amplitude (Fig. 1D) remained unchanged. All these ECG changes in the heart indicate the cardiac conduction abnormality.

### TLR4 activation increased inflammatory gene expression in rat heart and H9c2 cells

**Figure 2:**
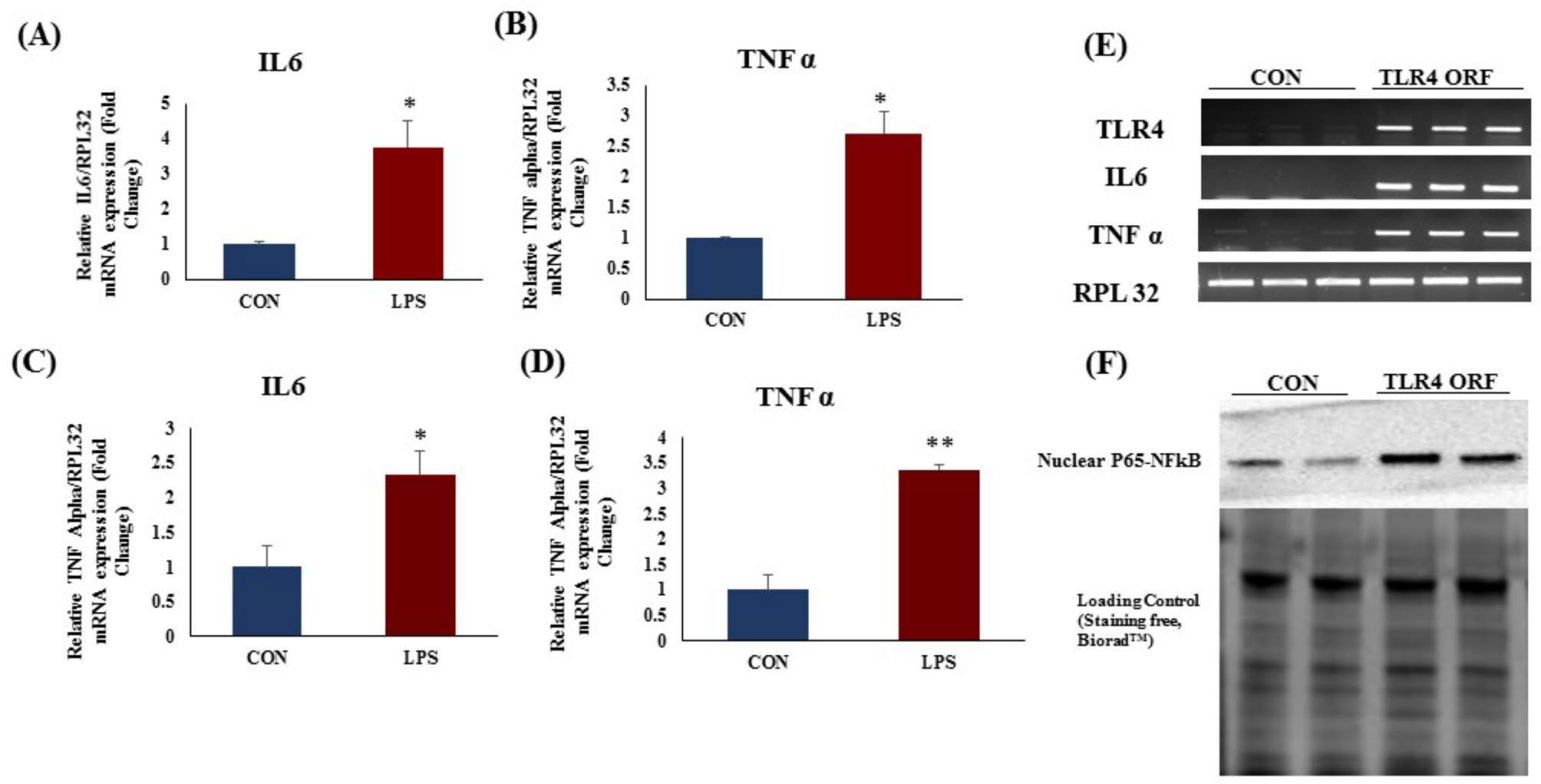
mRNA expression after TLR4 activation in rat heart and in H9c2 (LPS 1 µg/mL) cells. mRNA expression of (**A**) IL-6 (**B**) TNF-α in rat heart tissue. mRNA expression of (**C**) IL-6 (**D**) TNF-α in H9c2 cells. (**E**) mRNA expression of TLR4, IL-6 and TNF-α in TLR4 overexpressed H9c2 cells (**F**) NFkB p65 protein expression in TLR4 overexpressed H9c2 cells. The data was normalized to the expression of reference gene, *ribosomal protein L32* (RPL32). N = 4 for mRNA expression. *p < 0.05, **p < 0.01 vs CON group.

Myocardial mRNA expression of an important inflammatory genes TNF α and IL6 was significantly increased in LPS group as compared to CON group (Fig. 2A,B). TLR4 activation (Fig. 2C,D) as well as TLR4 overexpression (Fig. 2E,F) in H9c2 cells increased the mRNA expression of IL-6 and TNF alpha as compared to CON (p < 0.05).

### TLR4 activation increased myocardial fibrosis and fibrotic gene expression in rat heart

**Figure 3:**
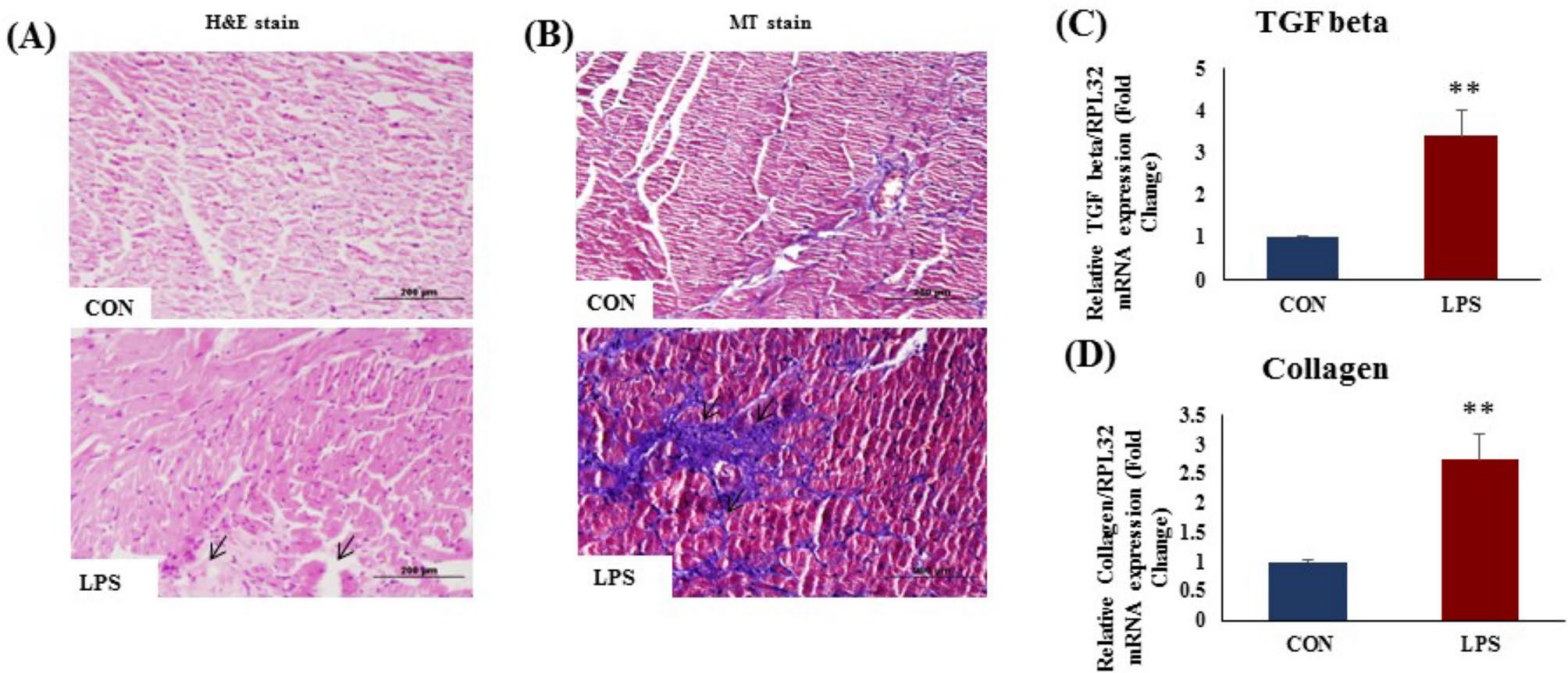
Cardiac cell phenotypes and histopathological examination in cardiac tissue and effect of TLR 4 activation; (**A**) Hematoxylin and eosin staining of rat heart tissue (**B**) Masson’s trichrome staining of rat heart tissue. (**C**) mRNA expression of TGF β (**D**) mRNA expression of collagen 1α in rat heart tissue. The mRNA expression data was normalized to the expression of reference gene, *ribosomal protein L32* (RPL32). Data shown as Mean ± SEM, (N = 4 for mRNA expression N = 3 for histopathology) *p < 0.05, **p < 0.01 vs CON.

The H&E stained sections of control (CON) rat hearts revealed normal cardiac morphology under a light microscope (Fig. 3A). In contrast, the cardiac histology of TLR4 activation group (LPS) showed discernible changes. Cardiomyocyte damage and loss was observed in LPS-treated hearts. There was a remarkable loss of normal arrangement of cardiomyocytes. Masson's Trichrome (MT) stained sections showed (Fig. 3B) prominent changes in the LPS-treated heart. Collagen deposition, a characteristic of replacement fibrosis, was significantly increased in LPS heart (Fig. 3B) as compare to CON. TLR4 activation significantly increased myocardial fibrosis (Fig. 3B) and mRNA expression of myocardial TGF-β (Fig. 3C) and collagen 1α (Fig. 3D) (p < 0.05) as compared to CON group (Fig. 3C).

### TLR 4 activation leads to mitochondrial dysfunction in heart

**Figure 4:**
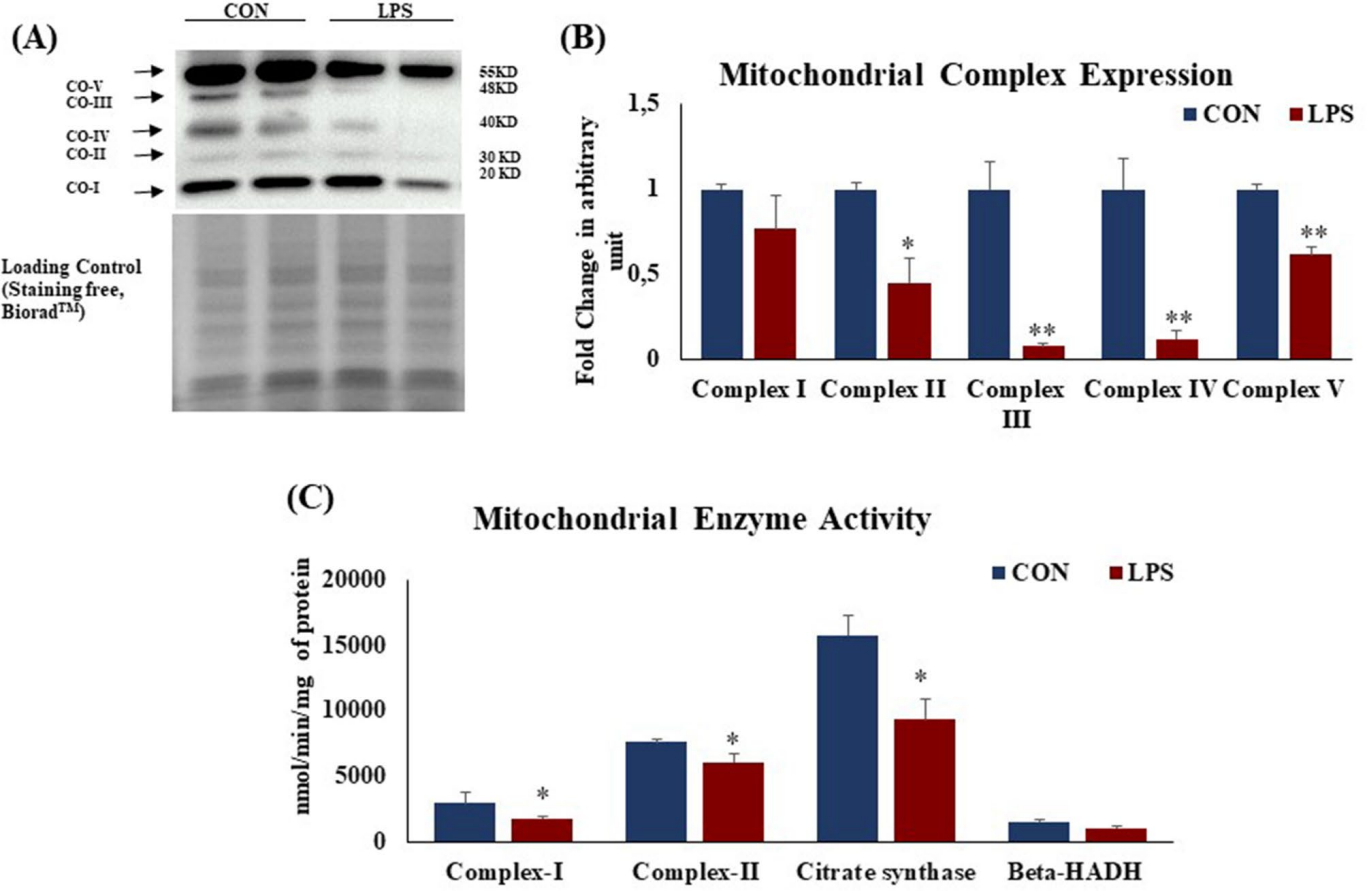
Protein expression of mitochondrial complexes in rat heart and effect of TLR 4 activation. (**A**) Protein expression of mitochondrial complexes; (I) Complex-I: NADH dehydrogenase (II) Complex-II: Succinate dehydrogenase (III) Citrate synthase (IV) β-hydroxy acyl coA dehydrogenase (V) ATP synthase. (**B**) Fold change in expression of mitochondrial complexes (I–V) in rat heart tissue. Whole gel stain was used for loading control. (**C**) Mitochondrial metabolic enzymes activity in rat heart and effect of TLR 4 activation. Data shown as Mean ± SEM, (N = 4 for enzyme activity and N = 3 for western blot) *p < 0.05, **p < 0.01 vs CON.

Protein expression of mitochondrial succinate dehydrogenase (complex II), cytochrome c oxidoreductase (complex III), cytochrome c oxidase (complex IV) and ATP synthase (complex V) (Fig. 4A,B) was significantly (p < 0.05) reduced in LPS group. Similar changes were observed in their enzyme activities. Myocardial enzyme activity of mitochondrial complex I, i.e. NADH dehydrogenase and complex II, i.e. succinate dehydrogenase (Fig. 4C) were reduced in LPS group as compared to CON group. Myocardial citrate synthase and 3-hydroxy-CoA dehydrogenase (Fig. 4C) activity was significantly (p < 0.05) reduced in LPS group.

### TLR4 activation induced oxidative stress in rat heart and H9c2 cells

**Figure 5:**
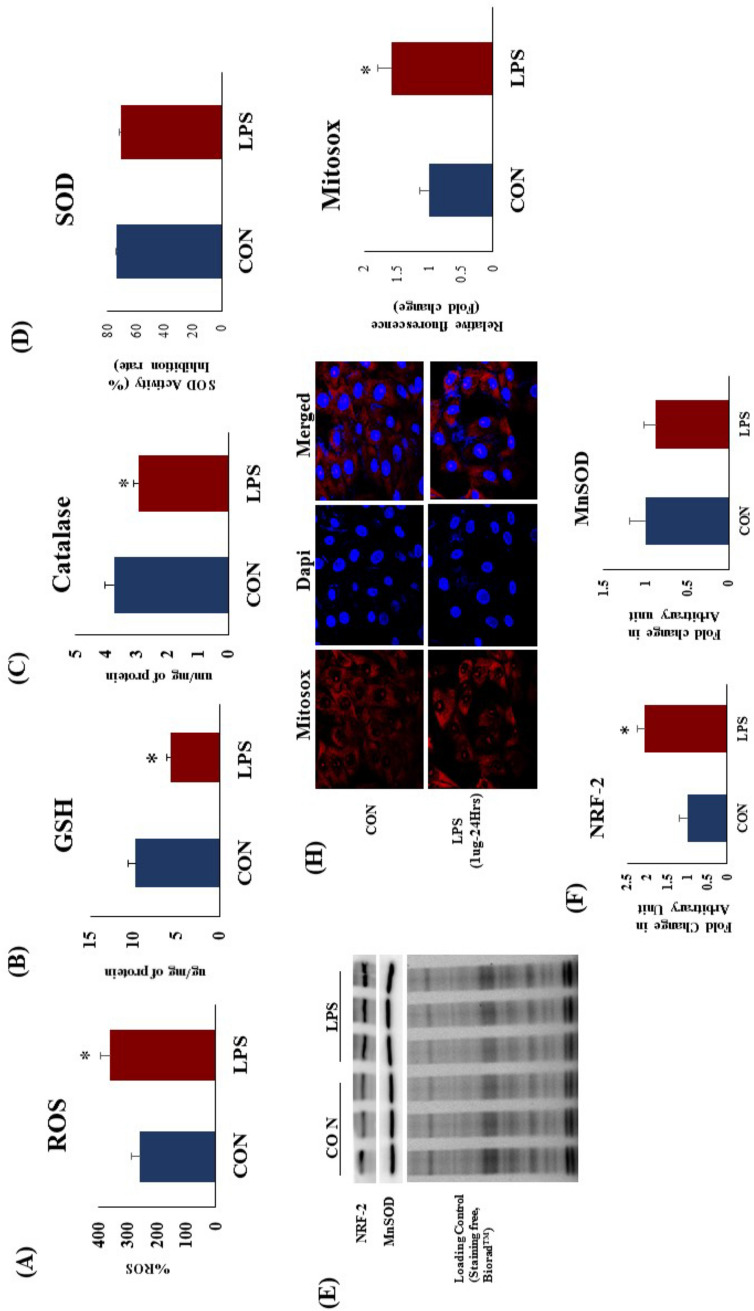
Cardiac redox status in heart and effect of TLR 4 activation on myocardial (**A**) ROS (**B**) Reduced glutathione (**C**) Catalase activity (**D**) SOD enzyme activity, (**E**–**G**) Protein expression NRF-2 and MnSOD. Whole gel stain was used for loading control. (**H**) Mitochondrial oxidative stress in H9c2 cells after TLR4 activation. Data shown as Mean ± SEM, (N = 3 for western blot, N = 5 for enzyme activity) *p < 0.05, **p < 0.01 vs CON.

After two weeks of TLR4 activation, there was a significant (p < 0.05) increase in cardiac ROS level (Fig. 5A). To confirm the finding in vitro, we demonstrated higher mitochondrial oxidative stress in TLR4 over expressed and TLR4 activated H9c2 cells (Fig. 5G,H). There was a significant increase in mitochondrial ROS in H9c2 cells after TLR4 activation as well as its over expression. There was a significant decrease (p < 0.05) in cardiac endogenous antioxidants like GSH content (Fig. 5B) and catalase activity (Fig. 5C) but not total SOD activity (Fig. 5D) in LPS treated hearts. TLR4 activation significantly increased NRF2 protein expression (Fig. 5D,E) which signifies the adaptive changes in heart to counteract TLR4 induced oxidative stress. However, MnSOD protein expression (Fig. 5D,F) was not decreased in LPS group as compared to CON.

### TLR4 activation increased apoptotic gene and protein expression in rat heart and apoptosis in H9c2 cells

**Figure 6:**
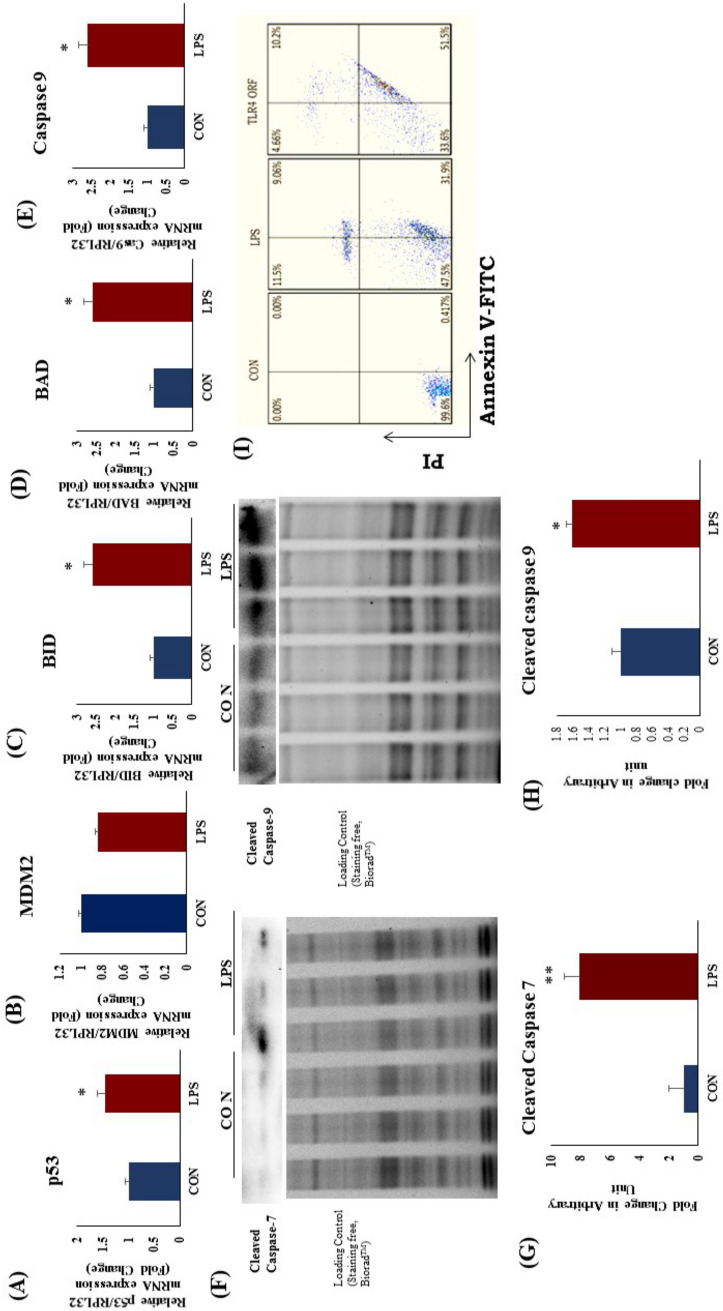
Apoptotic protein and gene mRNA expression in rat heart after TLR4 activation. (**A**) mRNA expression of p53, (**B**) mRNA expression of MDM2 (**C**) mRNA expression of BID (**D**) mRNA expression of BAD and (**E**) mRNA expression of caspase 9. (**F**) Protein expression of Cleaved caspase 7 and Cleaved caspase 9, (**G**) Fold change in expression of caspase 7, (**H**) Fold change in expression of caspase 9. (**I**) Apoptosis in H9c2 cells after TLR4 activation and overexpression. Whole gel stain was used for loading control for western blot. The mRNA expression data was normalized to the expression of reference gene, *ribosomal protein L32* (RPL32). (N = 3 for western blot, N = 4 for mRNA expression). *p < 0.05, **p < 0.01 vs CON group.

Expression of apoptotic genes p53, BAD, BID, Caspase-9 was significantly increased after LPS treatment as compared to CON (Fig. 6A,C,D). Protein expression of cleaved caspase-7 and cleaved caspase 9 was significantly high in LPS group as compared to CON (Fig. 6G). The change in cleaved caspase 3 was not found significant (Supplementary Fig. S8). To examine the effect of LPS treatment on H9c2 cells in vitro, we further evaluated the induction of apoptosis in presence of LPS. We found that, TLR4 overexpression and activation in H9c2 cells significantly increased the apoptosis as compared to CON (Fig. 6H). These results confirmed that, TLR4 activation increases cardiomyocyte loss in heart upon unmitigated activation of apoptosis.

### TLR4 activation increased apoptosis and p53 nuclear migration in rat heart and H9c2 cells

**Figure 7:**
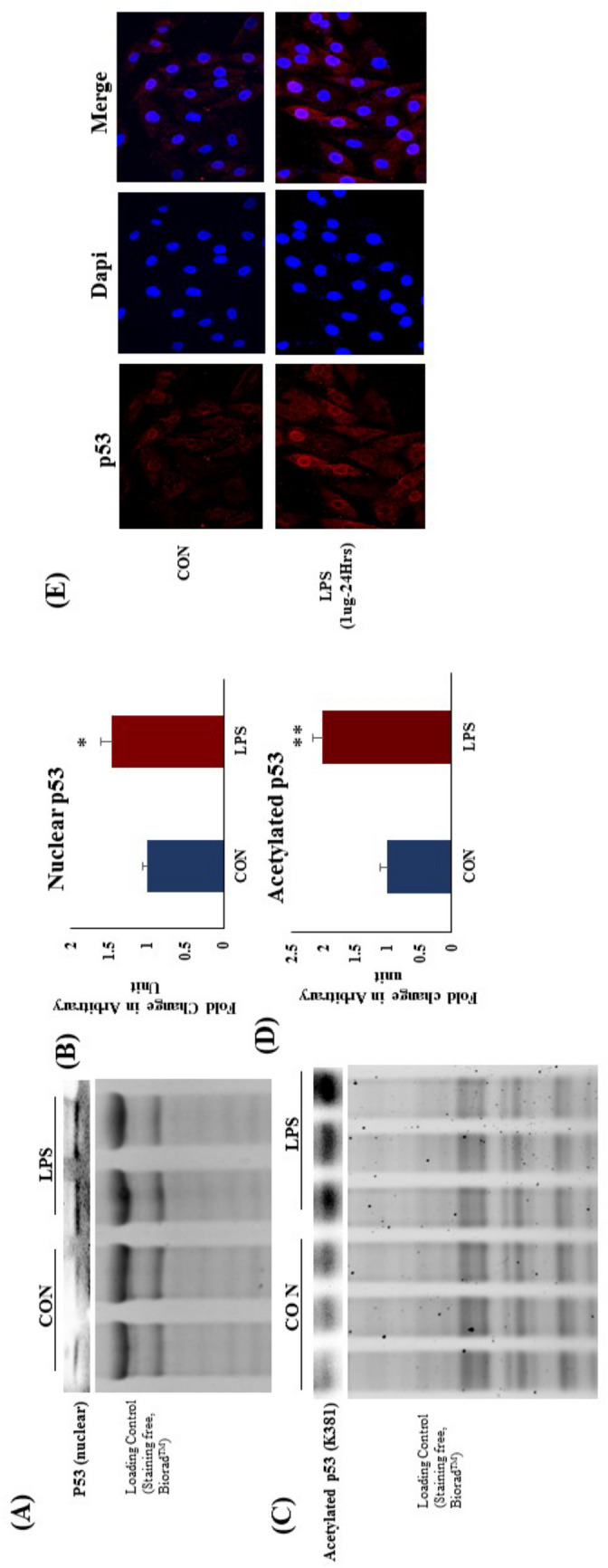
Protein expression and acetylation of p53 in rat heart and effect of TLR 4 activation. (**A**) Protein expression of nuclear p53, (**B**) Fold change in expression of p53 (**C**) Protein expression of acetylated p53, (**D**) Fold change in expression of acetylated p53 (**E**) Protein expression and nuclear translocation of p53 in H9c2 cells after TLR4 activation. Whole gel stain was used for loading control for western blot. (**G**) Data shown as Mean ± SEM, (N = 4 for confocal analysis and N = 3 for western blot) *p < 0.05, **p < 0.01 vs CON.

To evaluate the effect of increased p53 (K381) acetylation on its activity, we did protein expression analysis in cytoplasmic and nuclear fraction in rat heart after LPS treatment. We found that, p53 nuclear translocation was significantly increased in LPS group as compared to CON in rat heart (Fig. 7A). At the same time p53 gene expression was increased in LPS group (Fig. 6A). We validated the result in vitro and found similar effect on p53 nuclear translocation when H9c2 cells were activated with LPS. There was a significant increase in p53 expression and nuclear translocation after TLR4 activation in H9c2 cells (Fig. 7G).

### TLR4 activation increased p53 acetylation in rat heart

Acetylation of p53 in the cytoplasm is mainly governed by SIRT2. To evaluate the effect of SIRT2 downregulation after TLR4 activation, we analysed the acetylation status of p53. We found that, p53 acetylation (K381) was significantly increased in LPS group as compared to CON (Fig. 7C,D). It is reported that increased acetylation leads to increased binding affinity of p53 towards DNA. Hence, increased p53 acetylation increases the expression of several pro-apoptotic genes.

### TLR 4 activation decreased NAD^+^ level, and SIRT2 expression and its activity in rat heart

**Figure 8:**
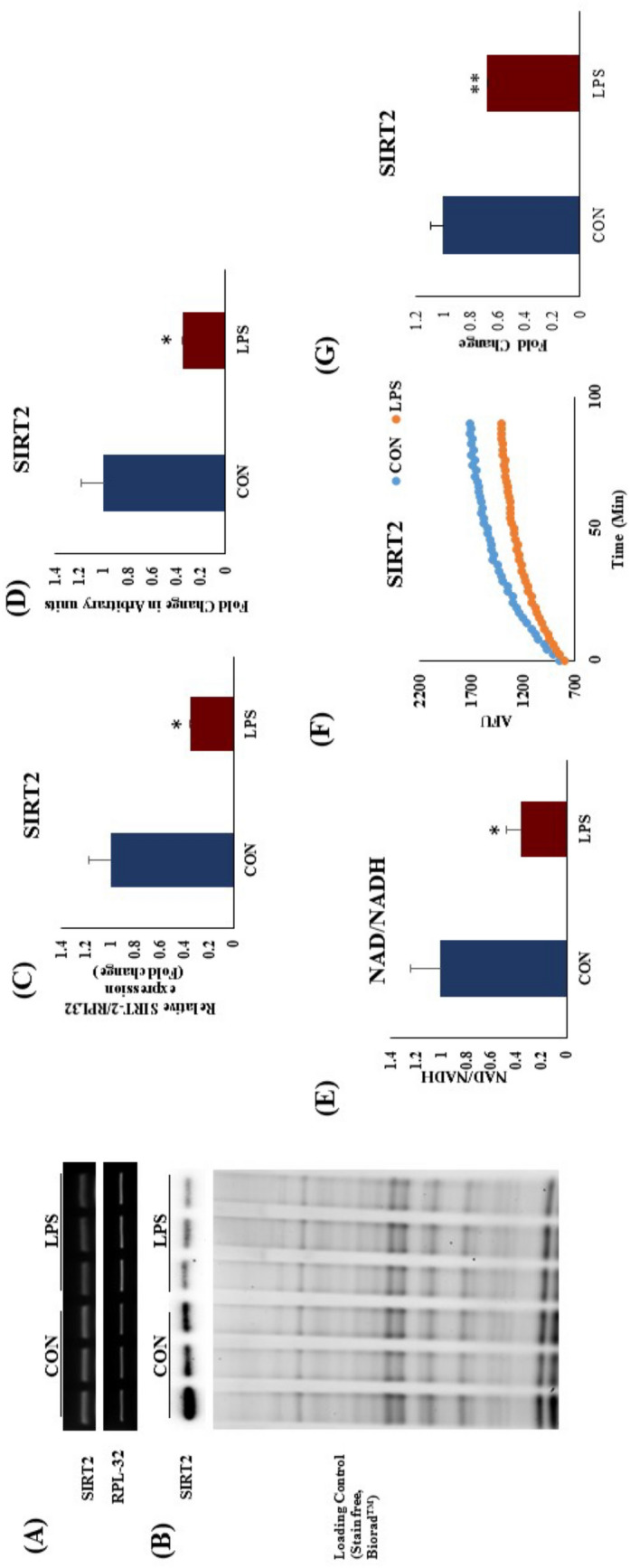
mRNA expression after TLR4 activation in rat heart. (**A**) mRNA expression of SIRT2, (**B**) Protein expression of SIRT2 (**C**) Fold change in mRNA expression of SIRT2 (**D**) Fold change in protein expression of SIRT2. Whole gel stain was used for loading control for western blot. The data for mRNA expression was normalized to the expression of reference gene, *ribosomal protein L32* (RPL32). (**E**) NAD^+^/NADH ration after TLR4 activation, (**F**) SIRT2 activity in rat heart after TLR4 activation. (N = 4 for mRNA expression and SIRT2 activity, N = 3 for western blot). *p < 0.05, **p < 0.01 vs CON.

To evaluate the role of NAD and SIRT2 in LPS induced apoptosis, we measured their level and enzymatic activity respectively, in rat heart tissue homogenate. We found that myocardial NAD^+^ level was significantly decreased in TLR4 activation group (Fig. 8E). Decreased NAD^+^/NADH ratio is a hallmark of perturbed cellular metabolism. We found that NAD^+^/NADH ratio was significantly decreased after LPS treatment (Fig. 8E). SIRT2 mRNA and protein expression was significantly decreased in LPS group as compared to CON (Fig. 8A,B). Our data suggest that, myocardial SIRT2 activity directly correlated with its expression and found to be decreased in LPS group as compared to CON (Fig. 8).

### SIRT2 over expression decreased p53 acetylation in H9c2 cells

**Figure 9:**
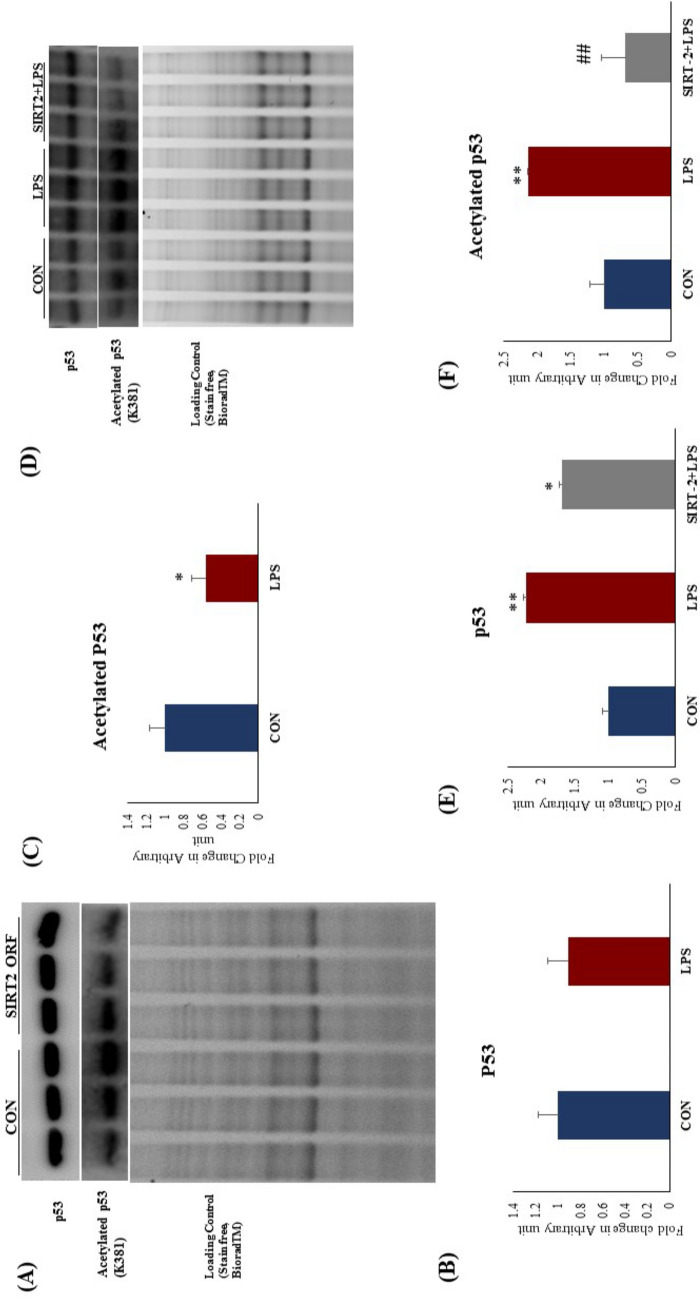
p53 protein expression and acetylation after TLR4 activation and SIRT-2 overexpression in H9c2 cells. (**A**) p53 protein expression and acetylation after SIRT-2 over expression in H9c2 cells (**B**) Fold change in protein expression of p53 (**C**) Fold change in p53 acetylation (**D**) p53 protein expression and acetylation after TLR4 activation and SIRT-2 overexpression (**E**) Fold change in protein expression of p53 (**C**) Fold change in p53 acetylation. Whole gel stain was used for loading control for western blot (N = 3 for western blot). *p < 0.05, **p < 0.01 vs CON, ^##^p < 0.01 vs LPS.

SIRT-2 mediated regulation of p53 acetylation is critical for its equilibrium between activated and inactivated state. SIRT2 over expression significantly reduced p53 acetylation in H9c2 cells (Fig. 9A,C). To evaluate the effect of SIRT-2 over expression after TLR 4 activation, we analysed the acetylation status of p53. We found that, TLR4 activation significantly increased p53 expression and acetylation (K381) in H9c2 cells. SIRT2 over expression successfully attenuated the TLR 4 mediated p53 acetylation (K381) in H9c2 cells. p53 acetylation (K381) was significantly increased in LPS group as compared to CON (Fig. 9D,E,F). Our data confirmed that SIRT2 was found to be critical for TLR 4 mediated p53 regulation.

## Discussion

In the present study, we have demonstrated that TLR4 is an important mediator for the development of cardiac fibrosis. It is well known that unmitigated TLR4 activation may lead to increased risk for cardiac inflammation. Many studies have shown that deletion of TLR4 may protect heart from ischemia reperfusion injury, cardiac hypertrophy and other cardiac complications^12,16^. A recent study showed that, repeated TLR4 activation during cardiac hypertrophy might lead to cardiac fibrosis and expression of extra cellular matrix proteins^15^. However, the exact mechanism responsible for the increased cardiomyocyte apoptosis and fibrosis after unmitigated TLR4 activation is not clear. We found that, TLR4 activation for a period of 14 days leads to increased cardiac apoptosis followed by increased expression of extra cellular matrix proteins and fibrosis. TLR4 activation also increased oxidative stress and mitochondrial dysfunction in heart**.**

We administered LPS through osmotic pump, which released LPS at a controlled rate i.e. 12.5 µg/kg/day for 14 days. Our objective here was to mimic the clinical condition of persistent activation of TLR4. This resulted in cardiac atrophy in LPS group. Heart weight-to-tail length ratio was found to be decreased in LPS group. This decrease in heart weight is due to the cardiomycyte death after LPS treatment. As cardiomyocytes are terminally differentiated cells and have a limited ability to regenerate, cardiomyocyte loss may cause serious adverse effect in cardiac physiology. In ECG analysis, QT interval was prolonged in LPS group, indicating conduction defects in these hearts. Apart from this, heart rate was found to be decreased in LPS group, which could be due to the depressive effect of TLR4 activation on heart. These research findings indicated that, activation of TLR4 has a remarkable adverse effect on cardiac structure and function.

As expected, there was a marked increase in inflammation, indicated by increased expression of TNF-α and IL-6 mRNAs in LPS treated heart. Being the major functional cells of heart, cardiomyocytes were the focus of our study. Cardiomyocyte inflammation was confirmed in H9c2 cells with increased expression of TNF alpha and IL-6 after LPS treatment. We observed similar results in TLR4-overexpressing H9C2 cells. mRNA expression of inflammatory genes such as TNF α and IL-6, and nuclear NFkB p65 protein expression was increased. This data indicates that, TLR4 regulates the expression of these inflammatory genes through NFkB p65 activation. Our in vitro mechanistic study supported the in vivo findings and suggest the critical role of TLR4 in cardiomyocyte inflammation.

Chronic cardiac inflammation and injury is followed by replacement fibrosis^17^. We observed cardiomyocyte damage and fibrosis in our histopathology study. There was a marked increase in TGF-β and collagen 1α mRNA expression, indicating fibrosis in LPS treated rat heart, due to loss of cardiomyocytes in these hearts. Increased fibrosis in heart causes hindrance for the action potential conduction through heart. This might underlie the disturbed ECG and prolonged QT. Overall these changes indicate the massive damage caused by TLR4 activation in the heart.

Cardiac damage is often associated with mitochondrial dysfunction and redox imbalance^18^. We found wide perturbations in the redox status and mitochondrial health of LPS-treated rat hearts. To evaluate the effect of TLR4 activation on mitochondrial function, we analysed the protein expression of electron transport chain (ETC). Mitochondrial protein expression of Complex-II, III, IV and V was significantly decreased in LPS heart as compared to control. Our data suggest that, decreased mitochondrial enzyme activity was the direct effect of TLR4 activation. Mitochondrial enzyme activity of complex I and II and citrate synthase, an important enzyme from TCA cycle were found to be significantly decreased in hearts in LPS treated rats. However, mitochondrial enzyme activity of 3-hydroxyacyl-CoA dehydrogenase (HADH), an important enzyme from beta-oxidation pathway was unchanged, indicating that TLR4 activation specifically altered oxidative phosphorylation in mitochondria, while sparing beta oxidation. We found that TLR4 over expression and activation in vitro in H9c2 cells increased mitochondrial oxidative stress significantly as compared to control. This shows the role of TLR4 in regulating mitochondrial function and redox balance, which is supported by previous literature.

To analyse the perturbations in redox status in rat hearts, we examined the level of reactive oxygen species (ROS) and lipid peroxidation as well as endogenous antioxidants like GSH and catalase. TLR4 activation significantly increased cardiac ROS level in LPS treated heart as compared to CON. Endogenous antioxidant status such as catalase activity and reduced glutathione level were significantly lower in LPS group. We found increased expression of cardiac NRF2 in LPS group, which implies the activation of adaptive mechanism to counterbalance oxidative stress in these hearts. However, protein expression of MnSOD, an important mitochondrial antioxidant in the cardiomyocytes was not decreased in LPS group.

To explore whether apoptosis pathway was activated in LPS treated hearts, we analysed the mRNA expression of apoptotic genes expression in heart. We found that, mRNA expression of BID, BAD and caspase 9 were increased in LPS group. In in vitro mechanistic study, we found that TLR4 activation in H9c2 cells increased the cellular apoptosis significantly. This result confirmed that, TLR4 activation was responsible for cardiomyocyte apoptosis in LPS treated hearts marked by increased apoptotic markers. While certain degree of apoptosis may occurs during normal physiology, TLR4-induced accelerated rate of cardiomyocytes death could be detrimental for heart as it has a limited capacity to regenerate. Therefore, blocking apoptosis in heart could be an attractive target to stop the progression of heart disease whereas cell death is prominent.

We further evaluated the molecular perturbations underlying the activation of apoptotic pathway, and found that, p53 mRNA expression was significantly increased in LPS group while MDM2 expression was unchanged. TLR4 activation significantly increased nuclear translocation of p53 in LPS group as compared to CON. p53 nuclear translocation is mainly governed by acetylation of p53 at lysine 381 (K381), which is a binding site for MDM2, a p53 inhibitor^19^. Acetylation of one or more lysine’s in a protein can have a functional effect on the protein by altering its conformation or its interaction with other proteins. Acetylation has an important effect of p53 function^19^. It has been reported that p53 acetylation increases its stability and transcription activity for apoptotic genes^20^. MDM2 binds at K381 to inhibit the p53 nuclear translocation. However, when p53 is acetylated at K381, MDM2 fails to bind p53 and hence results in increased nuclear translocation of the protein^19,20^. Phosphorylation of p53 is also reported to link to its nuclear translocation and required for p53-dependent apoptosis^21^. However, in the present study we have focused on acetylation of p53 and its regulation by sirtuins.

In nucleus, p53 acts as a transcription factor for the several apoptotic genes, which then further propagates an apoptotic cascade in the cardiomyocyte. SIRT2 is a cytoplasmic enzyme, which regulates p53 deacetylation, and hence its activity and nuclear translocation^22^. We found that, SIRT2 mRNA and protein expression was significantly decreased in LPS group as compared to CON. Subsequently we found that, SIRT2 activity was significantly decreased in LPS group. Next, we looked into the effect of decreased SIRT2 on p53 acetylation in LPS treated heart, and interestingly, p53 acetylation (K381) was significantly increased in LPS group. In mechanistic in vitro study we found that, SIRT2 overexpression significantly decreased p53 acetylation. TLR4 activation increased p53 acetylation in H9c2 cells, which was successfully attenuated by SIRT2 over expression. This finding confirmed that reduced activity of SIRT2 was actually responsible for increased acetylated form of p53 and its nuclear translocation. Investigators reported that SIRT2 is a repressor of the inflammatory process in brain microglial cells^23^ and inhibition of SIRT2 exacerbates neuroinflammation and blood brain barrier disruption in traumatic brain injury^24^. Recently, scientist have demonstrated that, SIRT2 is a key regulator of cell cycle and cell survival in cell^25^. TLR4 activation has long been known to play a critical role in inflammation. However, our findings elucidate the role of SIRT2-p53 axis in TLR4-mediated myocardial inflammation and apoptosis.

## Conclusion

TLR4 activation in rats promotes cardiac inflammation, mitochondrial dysfunction, apoptosis and fibrosis. SIRT-2 was found decreased in LPS group, which resulted in increased p53 acetylation followed by p53 nuclear translocation. p53 in the nucleus increased transcription of several apoptotic genes, which increased cardiomyocyte apoptosis in LPS group. SIRT2-p53 axis along with caspase 7/caspase 9 was found to play an important role in TLR4 mediated inflammation and apoptosis. Reducing TLR4 mediated fibrosis and apoptosis could be a novel approach in the treatment of heart diseases, where apoptosis plays an important role.

## Methods and materials

### Animal study

Male Sprague–Dawley rats (weighing 200–250 g) were procured from National Institute of Nutrition, Hyderabad, India. Rats were maintained at temperature 22 ± 2 °C, relative humidity of 40 ± 15% and 12-h dark/light cycle, in small animal facility (SAF) of Translational Health Science and Technology Institute, Faridabad, India. Animals had access to water and diet ad libitum throughout the experiment. All experimental procedures were performed with the approval of Institutional Animal Ethical Committee of Translational Health Science and Technology Institute, Faridabad, India, in accordance with the relevant guidelines and regulations.

### Drug solution preparation and dosing

Lipopolysaccharide (LPS) from *Escherichia coli* 0111:B4 strain (Invivogen) was used for TLR 4 activation^26^. LPS was dissolved in pyrogen-free saline and filled in ALZET mini-osmotic pumps for sustained release.

### Treatment schedule

After acclimatization to experimental conditions, rats were randomised into two groups: Control group (CON) and TLR4 agonist group (LPS). CON group was administered pyrogen-free saline, and LPS group was administered 12.5 µg/kg/day of LPS, through ALZET pumps. Animals were anaesthetized using a mixture of ketamine (75 mg/kg, IP) and xylazine (5 mg/kg, IP) for surgical insertion of ALZET pump. The treatment schedule was for a period of 14 days (N = 10). Food intake and body weight gain were monitored during the study. At the end of study, animals were euthanized, hearts were collected and stored in either – 80 °C or 10% phosphate-buffered formalin for downstream analysis.

### Cell culture

H9c2 rat cardiomyoblasts were purchased from ATCC (Manassas, VA), and cultured in Dulbecco’s modified Eagle medium (DMEM), supplemented with 10% fetal bovine serum and 100 μg/ml penicillin/streptomycin. Cell culture medium was replaced every 2–3 days, and the cells were sub-cultured for subsequent experimental procedures. On reaching 50–60% confluence, cells were treated with LPS (1 µg/ml) for 24 h. TLR4 and SIRT2 were over expressed using TLR4 c-DNA clone from Dharmacon, USA or SIRT2 c-DNA clone from Origene, USA respectively. Cells were transfected using Dharmafect transfecting reagent (Dharmacon, USA), as per the manufacturer’s protocol. After LPS treatment, cells were washed and processed for gene and protein expression analysis.

### Immunocytochemistry and confocal microscopy

H9c2 cells were seeded in six-well plates containing glass cover slips, in Dulbecco’s modified eagle's medium (DMEM) supplemented as described above. After 12 h, LPS (1 µg/ml) was added to the wells for 24 h, after which culture medium was removed. Cells were washed thrice with PBS and fixed in chilled methanol for 10 min. After blocking using 1% BSA solution for one-hour cells were incubated with anti-p53 (Abcam; ab131442) antibody overnight in a humidified chamber. Next, after three washes, cells were incubated with Alexa fluor 594 conjugated secondary antibodies (Thermo Fisher Scientific) for 2 h. Coverslips were then mounted using DAPI as a counterstain and viewed under a confocal imaging system (FluoView).

To quantify mitochondrial oxidative stress, cells were processed and fixed in methanol as described above, and incubated with MitoSOX (250 nM) for 10 min at 37 °C in the cell culture incubator. Subsequently, cells were washed twice with PBS and coverslips were then mounted using DAPI as a counterstain, and viewed under a confocal imaging system (FluoView). Confocal images were analyzed using Image J software for densitometric analysis.

### Flow cytometry for quantification of apoptosis

After LPS treatment, H9c2 cells were harvested, rinsed with PBS and centrifuged at 1000 × *g* for 5 min at 4 °C. This washing step was repeated twice. Subsequently, the cells were re-suspended in 500 μL binding buffer mixed with 5 μL Annexin V-FITC, and then kept in dark for 10 min at room temperature. Next, 5 μL of propidium iodide (PI) was added and the samples were again kept in darkness for 10 min at room temperature. Approximately 300 μL of binding buffer was added, and samples were analyzed in flow cytometer within 1 h. Cells of the control group without Annexin V-FITC and PI served as blank controls. Cells labeled with only PI were the PI single-labeled controls. Cells labeled with only annexin V-FITC were Annexin V-FITC single-labeled controls.

### Heart weight and tail length ratio

Rats were euthanized at the end of the experiment. Hearts were removed, washed in freshly prepared, ice-cold phosphate-buffered saline, dried on a blotting paper, and weighed. Body weight and tail length of all animals were measured right before euthanizing the animals. Heart weight/tail length ratio (mg/cm) was used to measure cardiac hypertrophy as described earlier^27^.

### Preparation of heart tissue homogenate

100 mg of rat heart tissue was homogenized in 2 mL 0.05 M phosphate buffer (pH 7.4), and centrifuged at 12,000 × *g* for 30 min at 4 °C to prepare heart tissue homogenate. From the resulting supernatant, aliquots were prepared and stored at − 80 °C for further analysis.

### Estimation of antioxidant parameters

Rat heart tissue homogenate was used to measure thiobarbituric acid reactive substances (TBARS). Supernatant from heart tissue homogenate was used to quantify reduced glutathione (GSH), reactive oxygen species (ROS) and catalase. While TBARS^28,29^ were measured as a marker of lipid peroxidation, GSH, CAT^30^ and SOD were estimated as endogenous antioxidants, as described before^31^.

### Isolation of mitochondria

Mitochondria isolation was performed from equal weight of heart tissues with mitochondria isolation kit (Pierce, Thermo Scientific, Cat No: 89801). Briefly, heart tissue was weighed, minced into small pieces, followed by homogenization using Dounce homogenizer. Mitochondria were then isolated from the tissue homogenate according to manufacturer’s protocol. Resultant mitochondrial pellet was resuspended in MTP buffer containing 110 mM mannitol, 60 mM Tris HCL, 60 mM potassium chloride, 10 mM dibasic potassium phosphate and 0.5 mM EDTA, pH-7.4. Mitochondrial purity and integrity were confirmed using Mito Tracker, as described before^32^.

### Mitochondrial respiratory chain complex activity in heart

The enzymatic activity of NADH-ubiquinone oxidoreductase (complex-I) and succinate-ubiquinone oxidoreductase (complex-II), two important enzymes from mitochondrial electron transport chain (ETC), was measured in the isolated mitochondria as previously described^33^. Activities of citrate synthase, an important enzyme of TCA cycle and, β-hydroxyacyl CoA dehydrogenase (Beta-HADH), an important enzyme for beta oxidation, were measured as per the protocol described previously^34,35^.

### Assessment of NAD/NADH in the diabetic heart

NAD and NADH levels in heart tissue homogenate were estimated using NAD/NADH Quantitation Kit (Promokine, Germany). Total NAD, NADH and NAD/NADH ratio were quantified as per formula mentioned in manufacturer’s protocol.

### Electrocardiogram recording

On 13th day of the experiment, ECG examination was performed. Rats were anaesthetized using a cocktail of ketamine (75 mg/kg, IP) and xylazine (5 mg/kg, IP) and kept in supine position on a homoeothermic blanket to ensure optimal body temperature. ECG was performed and data were analysed using PowerLab apparatus,with LabChart software, as described before^31^.

### Histopathology

Rat heart tissue was fixed in 10% phosphate-buffered formalin for 48 h. Formalin-fixed heart tissue was processed and embedded in paraffin. Paraffin sections (5 µm) were cut using microtome and mounted on glass slides. Hematoxylin and Eosin (H & E) staining was performed to examine the tissue morphology and damage. Masson’s trichrome staining (MT) was performed to examine the myocardial fibrosis. Stained sections were viewed under a light microscope as described previously^30,36^. Cardiomyocyte cell size from H & E stained sections and extent of cardiac fibrosis from Masson’s trichrome stained sections were quantified using ImageJ software, as described before^27,37^.

### Gene expression profiling

Rat heart tissue (n = 4) was processed using TRI reagent (Sigma Aldrich) to extract RNA, following manufacturer’s protocol. RNA was quantified using Nano Drop spectrophotometer (Thermo Scientific) and quality was assessed by RNA gel electrophoresis using 1% agarose gel prepared in DEPC-treated TBE buffer. RNA was treated with DNase and stored in -80 °C for future use. cDNA was synthesized from 2 µg RNA using superscript-III reverse transcriptase (Takara, USA). Real time polymerase chain reaction (RT-PCR) was carried out using SYBR Green mix (Takara, USA) on Light cycler 96 (Roche Inc., USA). Data for mRNA expression were normalized to the expression of reference gene *ribosomal protein L32* (RPL32)^38^.

### Immunoblotting

Rat heart tissue was processed using NE-PER kit (Thermo Scientific) to isolate nuclear and cytosolic proteins, according to manufacturer’s protocol. Tissue homogenates were centrifuged at 16,000 × *g* for 5 min at 4 °C, and supernatant (containing cytoplasmic extract) was transferred to pre-chilled tube. Pellet of remaining insoluble fraction was suspended in chilled nuclear extraction reagent by vortexing, and centrifuged at 16,000 × *g* for 10 min at 4 °C. Supernatant (containing nuclear extract) was transferred to pre-chilled tubes. Protein concentration was quantified using Pierce BCA Protein Assay kit (Thermo Scientific). Protein (25 μg) was resolved in 12% SDS–polyacrylamide gel (cast using TGX Stain-Free™ FastCast™ kit, Bio-Rad) and transferred to polyvinylidine difluoride (PVDF) membrane (GE Healthcare). Membrane was blocked using 3% non-fat milk in TBS-T (tris-buffered saline with 0.1% tween 20) at room temperature for 1hour, followed by incubation with appropriate primary antibody treatment overnight at 4 °C. Membrane was washed thrice with TBS-T for 5 min each, and then, incubated with corresponding HRP-labeled secondary antibody at room temperature for 1 h. Subsequently, membrane was washed thrice with TBS-T for 5 min each, and blot was visualized using Gel Doc XR system (Bio-Rad), using West Dura Pico (Thermo Scientific).

### Statistical analysis

All values are expressed as the mean ± standard error. Unpaired student t test was carried out to test for any differences between the mean values of the two groups. One way analysis of variance test followed by Bonferroni’s correction was performed to test the difference between mean values of more than two groups. Differences between groups were assumed significant if *p* < 0.05.

## Supplementary information

Supplementary Information.
