# Supplementary material for: Activation of toll like receptor 4 (TLR4) promotes cardiomyocyte apoptosis through SIRT2 dependent p53 deacetylation
Source: Sci Rep. 2020 Nov 6;10:19232. doi: 10.1038/s41598-020-75301-4 (PMC7648754; doi:10.1038/s41598-020-75301-4)
Supplement: Supplementary file 1 — Supplementary Information. [file 41598_2020_75301_MOESM1_ESM.docx]

**Activation of Toll Like Receptor 4 (TLR4) Promotes Cardiomyocyte Apoptosis through SIRT2 Dependent p53 Deacetylation**

**Parmeshwar Katare^1^, Hina Lateef Nizami^1^, Bugga Paramesha^1^, Amit K. Dinda^2^, Sanjay K. Banerjee^1, 3^.**

^1^Drug Discovery Research Centre (DDRC), Translational Health Science and Technology Institute (THSTI), Faridabad-121001, Haryana, India.

^2^Department of Pathology, All India Institute of Medical Sciences (AIIMS), Ansari Nagar, New Delhi- 110029, India.

^3^National Institute of Pharmaceutical Education and Research (NIPER), Guwahati- 781101, Assam, India.

*Corresponding to: Sanjay K Banerjee, Drug discovery research center, Translational Health Science and Technology Institute, Faridabad, Haryana, India. Tel: +9107042354595

Email addresses:skbanerjee@thsti.res.in, [banerjees74@hotmail.com](mailto:banerjees74@hotmail.com)

**Supplementary Information**

CON	TLR4 ORF

TNF α

CON	TLR4 ORF

CON	TLR4 ORF

CON	TLR4 ORF

NFkB

TLR4

IL-6

RPL-32

Loading control for NfKB

CON	TLR4 ORF

CON	TLR4 ORF

**Supplementary Figure S1. Uncropped agarose gel and western blot.** Uncropped agarose gel for Fig. 2E and uncropped western blot and loading control 2F. Boxes indicate cropped area.

Oxophos

Loading control

CON	LPS

CON	LPS

**Supplementary Figure S2. Uncropped Western blots.** Uncropped Western blots and loading control for Fig. 4a. Boxes indicate cropped area.

NRF-2

SOD2 (exposure-75 sec)

CON	LPS

SOD2 (exposure-60 sec)

Loading control NRF-2, SOD2 and cleaved caspase-7

CON	LPS

CON	LPS

CON	LPS

**Supplementary Figure S3. Uncropped Western blots.** Uncropped Western blots for Fig. 5E and loading control for NRF-2, SOD2 and cleaved caspase-7 (Fig. 6F). Boxes indicate cropped area.

Loading control Cleaved caspase-9

Cleaved Caspase-7

Cleaved Caspase-9

CON	LPS

CON	LPS

**Supplementary Figure S4. Uncropped Western blots.** Uncropped Western blots and loading control for Fig. 6F. Boxes indicate cropped area.

CON	LPS

Acetylated p53 (K381)

P53 (Nuclear)

CON	LPS

Loading control Acetylated p53

Loading control for p53

CON	LPS

CON	LPS

**Supplementary Figure S5. Uncropped Western blots.** Uncropped Western blots and loading control for Fig. 7A and 7C. Boxes indicate cropped area.

SIRT-2

SIRT-2

CON	LPS

CON	LPS

RPL-32

CON	LPS

SIRT-2

Loading control for SIRT-2

CON	LPS

**Supplementary Figure S6. Uncropped Western blots.** Uncropped agarose gel image, Western blots and loading control for Fig. 8A, 8B. Boxes indicate cropped area.

SIRT-2

RPL-32

CON	SIRT-2 ORF

CON	SIRT-2 ORF

**Supplementary Figure S7. SIRT-2 mRNA expression in H9c2 cells after SIRT-2 over expression.** Uncropped agarose gel image. Boxes indicate cropped area.

Loading control

CON	LPS

Cleaved caspase 3

CON	LPS

**Supplementary Figure S8.** Cleaved caspase 3 expression in rat heart after TLR4 activation. **Uncropped Western blots and loading control.** Uncropped Western blots Fig. S8. Boxes indicate cropped area.

Loading control

CON	LPS

CON	LPS

Phospho NFkB (p65)

**Supplementary Figure S9.** Phospho NFkB (p65) protein expression in rat heart after TLR4 activation. **Uncropped Western blots and loading control.** Uncropped Western blots Fig. S9. Boxes indicate cropped area.

Acetylated P53

CON	SIRT-2 ORF

P53

CON	SIRT-2 ORF

Loading control

CON	SIRT-2 ORF

**Supplementary Figure S10.** P53 protein expression and acetylation. **Uncropped Western blots.** Uncropped Western blots and loading control Fig. 9A. Boxes indicate cropped area.

CON	LPS	SIRT-2 ORF+LPS

CON	LPS	SIRT-2 ORF+LPS

Acetylated P53

P53

Loading control

CON	LPS	SIRT-2 ORF+LPS

**Supplementary Figure S11.** P53 protein expression and acetylation after TLR4 activation in H9c2 cells. **Uncropped Western blots.** Uncropped Western blots and loading control Fig. 9D. Boxes indicate cropped area.

**Supplementary Figure S12.** Schematic representation of proposed mechanism of TLR4 induced cardiac inflammation, apoptosis and fibrosis.
